# Auditory Same/Different Concept Learning and Generalization in Black-Capped Chickadees (*Poecile atricapillus*)

**DOI:** 10.1371/journal.pone.0047691

**Published:** 2012-10-15

**Authors:** Marisa Hoeschele, Robert G. Cook, Lauren M. Guillette, Allison H. Hahn, Christopher B. Sturdy

**Affiliations:** 1 Department of Psychology, University of Alberta, Edmonton, Alberta, Canada; 2 Department of Psychology, Tufts University, Medford, Massachusetts, United States of America; 3 Centre for Neuroscience, University of Alberta, Edmonton, Alberta, Canada; Duke University, United States of America

## Abstract

Abstract concept learning was thought to be uniquely human, but has since been observed in many other species. Discriminating *same* from *different* is one abstract relation that has been studied frequently. In the current experiment, using operant conditioning, we tested whether black-capped chickadees (*Poecile atricapillus*) could discriminate sets of auditory stimuli based on whether all the sounds within a sequence were the *same* or *different* from one another. The chickadees were successful at solving this same/different relational task, and transferred their learning to same/different sequences involving novel combinations of training notes and novel notes within the range of pitches experienced during training. The chickadees showed limited transfer to pitches that was not used in training, suggesting that the processing of absolute pitch may constrain their relational performance. Our results indicate, for the first time, that black-capped chickadees readily form relational auditory *same* and *different* categories, adding to the list of perceptual, behavioural, and cognitive abilities that make this species an important comparative model for human language and cognition.

## Introduction

Concept formation was originally thought to depend on language and thus be a potentially unique human trait ([1]; recent reviews: [2]–[3]). The capacity to form concepts, however, is now acknowledged as a process that commonly exists across the animal kingdom [4]. Many researchers have subsequently argued that the capacity for abstraction is an essential component of language rather than the reverse. In other words, that abstraction is required for language rather than that language is required for abstraction. For example, in their discussion of how linguistics can be studied from an interdisciplinary perspective, Hauser, Chomsky, and Fitch [5] suggest that a conceptual-intentional system is one of the broad systems necessary for language. By isolating and studying conceptual-intentional features, we may thus be studying an important building block of the human language faculty. Cross-species study of these potentially-important building blocks can help us uncover what Hauser et al. refer to as the *narrow faculty of language*. The narrow faculty of language refers to the critical component(s), yet undefined, which separates human language from similar abilities in other animals.

Hauser, Chomsky, and Fitch [5] also argue that another important piece of the language puzzle is an appropriate sensory-motor system for supporting language. Studying the conceptual-intentional system that is essential for language is therefore of particular interest in vocal-learning animals that may already have a language-relevant sensory-motor architecture. Specifically, vocal learners produce vocalizations based on their experience with other vocalizing conspecifics, and require this experience to produce at least some species-typical vocalizations. This is a relatively rare ability so far found in only humans, oscine songbirds, parrots, hummingbirds, cetaceans, bats, and elephants [6]. Our study species, the black-capped chickadee (*Poecile atricapillus*), belongs to the oscine songbirds, one of the few groups that learn their vocalizations from a tutor [7]. Songbird vocal learning is analogous to human vocal learning in terms of behavioural stages, neurobiology, and genetics [7]–[8]. In addition, at least some vocal-learning species appear to have other relevant sensory-motor abilities that may be tied to language, such as rhythmic entrainment [9]–[10].

Avian vocal learners are especially important to examine with respect to the learning and use of conceptual relations because of their frequently greater reliance on the use of absolute features in learning and performing discriminations. Songbirds in particular are known for their highly-precise absolute pitch abilities compared to mammals [11]. Because of this greater attention to absolute factors, it appears that this predisposition may limit their abilities to learn relational discriminations. For instance, European starlings (*Sturnus vulgaris*), trained to discriminate note sequences with a simple relative pitch rule, were unable to transfer this discrimination to a novel range of pitches without considerable retraining, tending instead to memorize the absolute pitch of notes within the sequences [12]–[13]. In recent work studying auditory sequence patterns, Comins and Gentner [14] found that, although it was possible to train starlings to use relative position under ideal conditions and with extensive training, starlings primarily used the absolute position of sounds in a serial sequence to classify them. In the same vein, chickadees often have difficulty with relative pitch discriminations [15]–[17], while they do well at absolute pitch discriminations [18]. In the non-vocal learning pigeon, the use of absolute rather than relational strategies to solve problems has been observed across several stimulus modalities. For example, spatial research reveals that pigeons often appear to use absolute rather than relational strategies to locate a target, although they can code relational cues and can use them in specific cases, such as when there are no global orienting cues (see [19] for discussion). A similar tendency to first encode absolute, but later encode and use relational features, was also seen with pigeons in an auditory task where both of these properties were simultaneously available [20].

**Figure 1:**
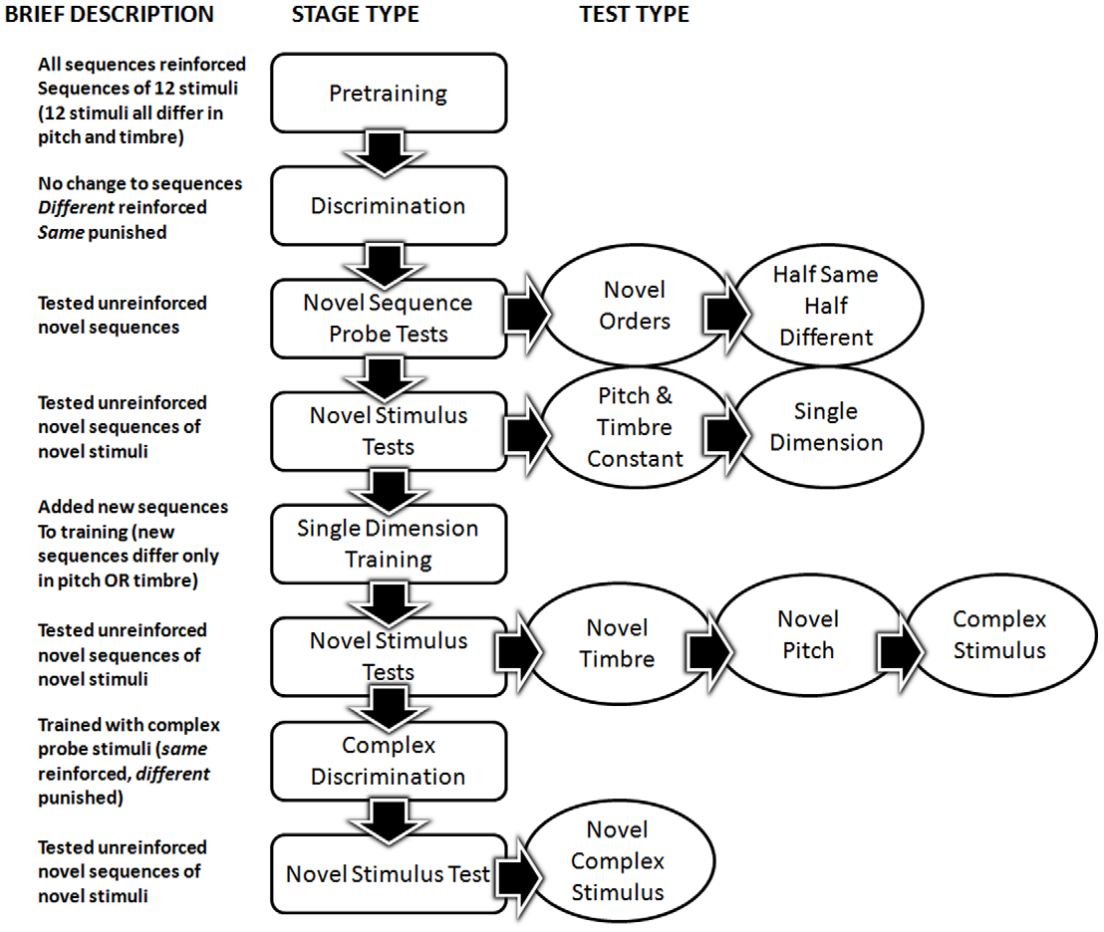
Flowchart of the experimental procedure. The boxes show the different stages with short titles. Descriptions to their left give more information. All probe stages show the order of test sessions in bubbles to the right.

Nevertheless, a growing literature has established that nonhuman primate and avian species can learn relational discriminations that cannot be solved using only absolute stimulus features (e.g., [21]–[24]). One form of abstract concept formation that has received a great deal of attention is the same/different (S/D) relation. In S/D discriminations, subjects assess whether items presented together are the *same* or *different*. By using members of the same set of items as components of both the *same* and *different* stimulus combinations, subjects cannot use absolute or item-specific features to categorize the stimulus. Instead, the animals must learn to judge the relations between the items. Of most importance to the idea of the animals learning about conceptual relations is that such S/D learning can be shown to transfer to novel items, suggesting that the animals learned to abstract these relations in order to classify them. For instance, pigeons have been shown to be able to acquire and transfer S/D discriminations in both the visual (e.g., [22], [25]–[28]) and auditory domains [20], [29].

**Table 1:** Note names and fundamental frequencies (f0) of the pitches used in this experiment.

Note Name	f0	Experimental Use
C3	131	Training
C^#^3/D^b^3	139	Within Probe
D3	147	Within Probe
D^#^3/E^b^3	156	Training
E3	165	Training
F3	175	Within Probe
F^#^3/G^b^3	185	Within Probe
G3	196	Training
G^#^3/A^b^3	208	Training
A3	220	Within Probe
A^#^3/B^b^3	233	Within Probe
B3	247	Training
C4	262	Between probe
C^#^4/D^b^4	277	Between probe
D4	294	Between probe
D^#^4/E^b^4	311	Between probe
E4	330	Between probe
F4	349	Between probe
F^#^4/G^b^4	370	Between probe
G4	392	Between probe
G^#^4/A^b^4	415	Between probe
A4	440	Between probe
A^#^4/B^b^4	466	Between probe
B4	494	Between probe
C5	523	Within Probe
D5	587	Training
D^#^5/E^b^5	622	Within Probe
E5	659	Within Probe
F5	698	Training
F^#^5/G^b^5	740	Training
G5	784	Within Probe
G^#^5/A^b^5	831	Within Probe
A5	880	Training
A^#^5/B^b^5	932	Training
B5	988	Within Probe
C6	1047	Training

In the current study, we assessed whether black-capped chickadees could successfully learn an auditory S/D discrimination and whether they could transfer this discrimination to novel stimuli. Auditory S/D discriminations are likely to play an important role for chickadees in the wild. For example, black-capped chickadees establish and defend territories in the spring using their *fee-bee* song which can be sung at a range of absolute pitches. One way of demonstrating increased aggression is by matching the *fee-bee* frequency recently used by a nearby male. Based on social factors, such as whether they are similar in dominance rank or whether they are flock mates, chickadees are more or less likely to match [30]. This capacity to match requires the recognition and discrimination of how similar the songs are to each other. Black-capped chickadees also appear to have relative pitch cues within their song to which other members of the species attend. Males will respond with more territory defense to playbacks of natural song than songs altered in relative pitch and females will similarly respond more sexually to natural songs than those altered in relative pitch [31]. Another possible example of S/D abilities playing a role in natural behaviour arises when multiple chickadee species live sympatrically. Chickadees use their namesake *chick-a-dee* call to coordinate movement among flock mates [32]. All chickadee species produce this call, but there are acoustic differences among species in the production of the call [33]–[38]. In order to respond appropriately to any one instance of a *chick-a-dee* call, a chickadee needs to be able to discriminate whether the call comes from the same species as itself (a conspecific) or a different species (a heterospecific; e.g., [39]).

**Figure 2:**
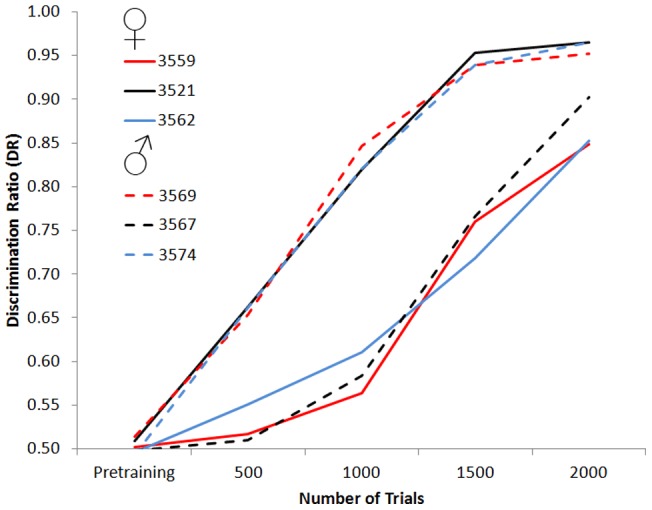
Discrimination ratio(DR) to *same* and *different* sequences during discrimination. The DR was calculated for blocks of 500 trials during sessions where S+ s were reinforced with a probability of 100%. A DR of zero indicates responding only to the S− *same* category, a DR of 0.50 indicates equal responding to both categories, and a DR of 1.00 indicates responding only to the S+ *different* category (see response measures section for details on calculation). The *x* axis shows the number of trials. The last block of *pretraining* data, where *same* and *different* sequences are not differentially reinforced, is also included as comparison. Female chickadees are shown with solid lines, male chickadees are shown with dotted lines.

We trained six chickadees to complete a S/D discrimination by training them to respond to *different*, but not *same*, auditory sequences using a procedure similar to that previously used with pigeons [29]. We chose to study auditory stimuli because of their relevance in vocal learning, and thus in the sensory-motor aspect of language-learning properties. As vocal learners, audition is also of high importance for black-capped chickadees in the wild, given that they use acoustic communication for mate attraction, territory defense, flock cohesion and predator mobbing among other things [40]. After learning the discrimination, we subsequently tested the chickadees with novel stimuli using non-reinforced probe trials to test for concept formation. Because of their capacity for vocal learning and greater attention to auditory stimuli, we predicted and subsequently found that black-capped chickadees would discriminate *same* from *different* to a higher level of discrimination and more rapidly than pigeons tested previously under similar conditions, and that they would be able to readily transfer this discrimination to novel stimuli.

**Figure 3:**
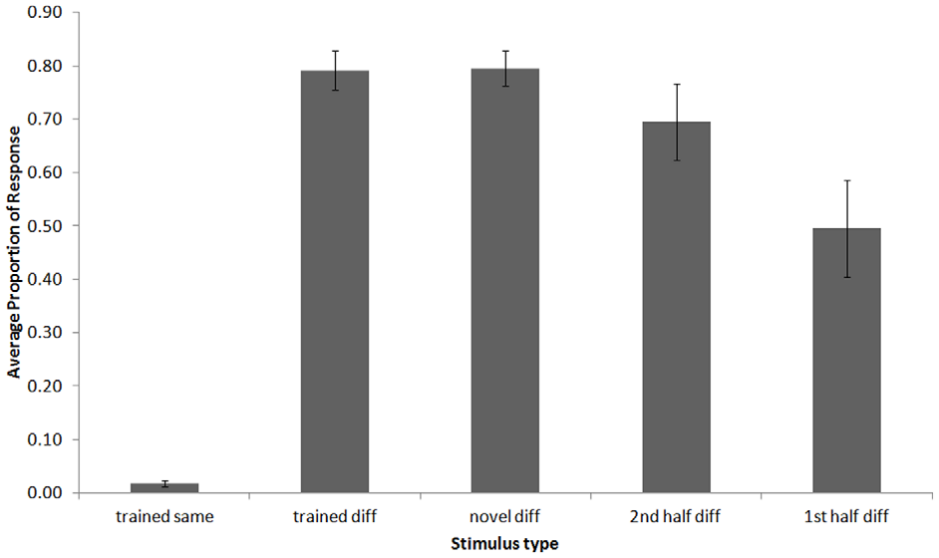
Average proportion of response during novel sequence probes. These tests used novel sequences of the stimuli presented in training only. In other words, only stimulus order was novel in these tests, not the sounds themselves. Error bars represent standard error.

## Methods

### Animals

Six wild-caught black-capped chickadees, three males and three females, were tested. Chickadees were captured in either Edmonton, Alberta (53°34′N, 113°31′W) or from an acreage outside of Stony Plain, Alberta (53°31′N, 114°00′W). All chickadees were determined at the time of capture to be at least one year of age as determined by the colour and shape of their outer tail retrices [41]. Prior to the experiment, chickadees were individually housed in Jupiter Parakeet cages (30×40×40 cm; Rolf C. Hagen, Inc., Montreal, Canada) in colony rooms on a light cycle that approximated the natural amount of daylight for the Edmonton region. The chickadees had visual and auditory, but not physical, contact with one another. All chickadees had *ad libitum* access to food (Mazuri Small Bird Maintenance Diet; Mazuri, St. Louis, MO), water (with added vitamin supplement on alternate days; Hagen, Rolf C. Hagen, Inc., Montreal, Canada), grit, and cuttle bone. To ensure good health, this diet was supplemented with a meal or super worm three times a week, an egg and greens mixture (spinach or parsley) twice a week, and 3–5 sunflower seeds daily.

**Figure 4:**
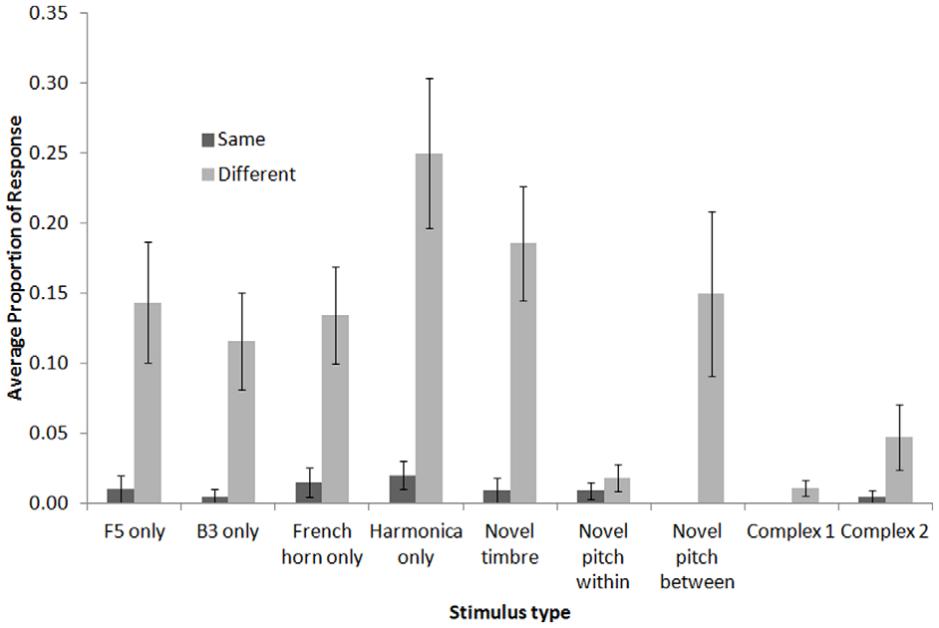
Average proportion of response during novel stimulus probes. For each novel stimulus type, we generated novel *same* (black bar) and novel *different* (grey bar) sequences. Error bars represent standard error.

Throughout the experiment, chickadees were housed in individual operant chambers (see below), maintained on the natural light cycle with *ad libitum* access to water, grit and cuttle bone. Supplemental meal or super worms were provided once in the morning and once in the afternoon. During experimentation, however, food was only available as a reward for correct responding in the operant discrimination task. Each chickadee had prior experience with auditory discriminations involving natural and synthetic stimuli (natural or synthetic *fee-bee* songs; [17], or *chick-a-dee* call note stimuli; [42]), but were naïve to the current stimulus set.

**Figure 5:**
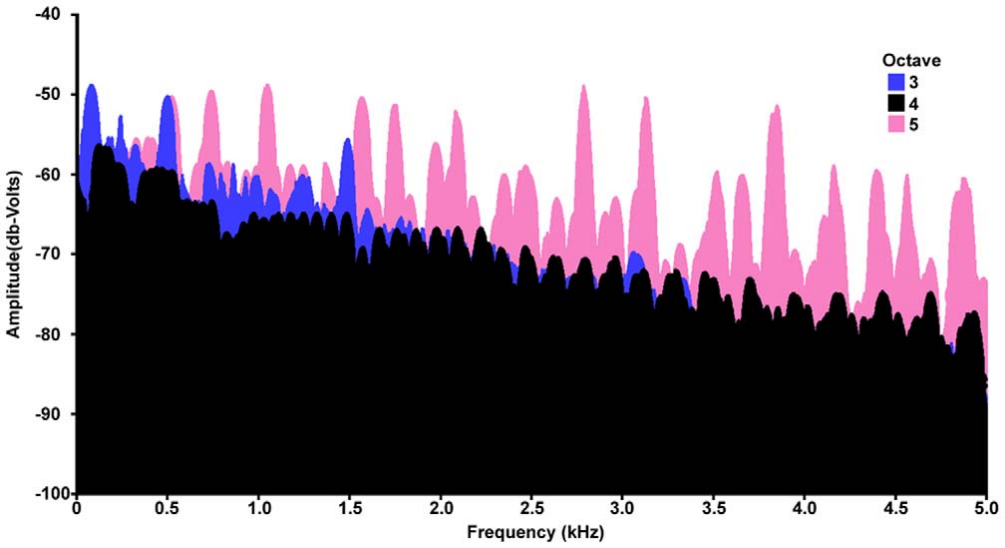
Maximum amplitude across training and novel pitch stimuli separated by the octave of the fundamental frequency. Both training and probe stimuli are included from the novel pitch stimulus probe. Frequencies from 0–5000 Hz are presented as the majority of the spectral frequencies occurred within this range. The three octaves are presented separately, however, each timbre and note within each octave are presented together, thus each line represents the maximum across all notes within that octave. Notes generated in octaves three and five were used for training and novel pitch within probe stimuli, and octave four was used for novel pitch between probe stimuli.

This study was conducted in accordance with the Canadian Council on Animal Care Guidelines and Policies with approval from the Animal Care and Use Committee for Biosciences for the University of Alberta and the Life and Environmental Sciences Animal Care Committee for the University of Calgary Life. Chickadees were captured and research was carried out under an Environment Canada Canadian Wildlife Service Scientific permit, Alberta Fish and Wildlife Capture and Research permits, and City of Edmonton Parks Permit. Chickadees were monitored throughout the day and provided with worms twice daily during for the duration of the experiment and given free-feeding days during shape training to ensure their welfare and good health. All chickadees were returned to the colony room upon completion of the experiment to be used in future experiments.

### Apparatus

During the experiment, the chickadees lived in modified colony room cages (30×40×40 cm) that served as operant testing chambers. Each cage was housed in separate, ventilated, sound-attenuating chambers. Each cage had three perches and dispensers for water and grit. The chambers were illuminated by a 9-W, full-spectrum fluorescent bulb. An opening on the side of the cage (11×16 cm) gave each chickadee access to a motor-driven feeder that delivered food [43]. Both the feeder and the perch closest to the feeder (the “request perch”) had infrared cells to monitor the chickadees' position. A computer and single-board computer [44] scheduled trials and recorded responses to stimuli. Stimuli were played from a CD through an integrated amplifier (either a Cambridge A300, Cambridge Audio, London, England or an NAD310, NAD Electronics, London, England) and a full-range speaker (FE108Σ, Fostex Corp., Japan; frequency range 200–16,000 Hz) located beside the feeder.

### Stimuli

All stimuli were 16-bit, 44,100 Hz, synthesized sounds created using Sonar sound synthesis software (Cakewalk, Boston, USA). All stimuli were broadcast at between 75–80 dB as measured from the chickadee's typical head position from the request perch using a sound level meter (A weighting, slow; Radio Shack, Fort Worth, TX, USA). For initial training, the sound sequences were made from 12 stimulus notes (C3-piano, D#3-guitar, E3-vibraphone, G3-reed organ, G#3-violin, B3-harmonica, A5-alto sax, A#5-oboe, D5-trumpet, F5-french horn, F#5-flute, C6-clarinet). Thus, six of these notes were the 3^rd^ octave and six were from the 5^th^ & 6^th^ octave. Each stimulus had a different pitch and timbre and thus the differences among stimuli were redundant across these two dimensions. All stimuli were 500 ms in duration with five ms tapers at each end to avoid transients. Sequences of 12 stimuli were assembled with silent inter-stimulus-intervals (ISI) of 100 ms. *Same* sequences consisted of one stimulus repeated 12 times (i.e., same pitch and same timbre) and *different* sequences consisted of all 12 stimuli each played once (i.e., all notes had a different pitch and timbre). *Different* sequences were created such that all notes appeared in each ordinal position an equal number of times using a Latin square.

### Procedure

We have provided a summary of the procedural design in Figure 1. The details about each stage of the experiment are described below.

#### Pretraining

After a chickadee learned to use the request perch and feeder, experimental pretraining began. Trials were available continuously throughout the day and data were collected in 504-trial blocks. Blocks not completed at the end of the day were resumed at the start of the following day. Trials were initiated by landing on the request perch and remaining for one second on average (randomized between 0.9–1.1 s). In pretraining, only 12 stimuli were used to make up all the sequences. *Same* sequences were a sequence of one of the 12 stimuli repeated 12 times. *Different* sequences had all 12 stimuli in a random order. During each trial, a sequence was randomly chosen without replacement from the pool and played through the speaker. During this stage, we tested all 12 possible *same* sequences and a randomly predetermined set of 252 possible *different* sequences (each of the 12 stimuli occurred in each position 21 times as determined by 21 unique 12×12 Latin squares). Chickadees were trained in 504 trial blocks where each possible *same* sequence occurred 21 times such that *same* and *different* sequences each made up half the trials. Within a block, a sequence was selected from the 504 possible sequences without replacement. This process was repeated until all sequences were presented, and the cycle repeated.

If a chickadee left the perch during the playback of a sequence (i.e., an interrupted trial), the house lights turned off for a 30-s delay during which no new trial could begin. Training with these interrupted trials ensured that a chickadee was presented the entire sequence before making a response. If the chickadee entered the feeder within one second after the completion of the sequence (*same* or *different* sequence), the chickadee received 1-s access to food. This was followed by a 30-s inter-trial interval (ITI) with the house light on. If the chickadee left the perch upon completion of the sequence, but did not enter the feeder, the trial ended after one s and the chickadee could then initiate a new trial. A new trial could be initiated by either leaving and returning to the request perch or waiting for a 60-s ITI. Pretraining continued until each chickadee was going to the feeder on at least 60% of the trials for at least six 504-trial blocks and responding within ≤3% difference to the future S+ and S− sequences for at least four blocks. This ensured there were no initial response biases to either *same* or *different* sequences.

#### Discrimination training

Discrimination training was identical to pretraining, except that chickadees were differentially rewarded for entering the feeder only following *different* sequences (S+). If they entered the feeder after a *same* sequence, then the lights were turned off for a 30-s delay (S−). This discrimination training continued until a chickadee completed six 504-trial blocks (the last two occurring consecutively) in which the discrimination ratio (DR) was greater than or equal to 0.80 (see response measures, below, for DR calculation).

At this point, the percentage of trials with food reward following *different* S+ sequences was reduced to 85%. On non-reinforced S+ trials, the chickadee received a 30-s ITI with the house light on, but had no access to food. This was done so that the chickadees experienced trials that were neither rewarded nor punished in preparation for later probe trials. Chickadees were trained with reduced reinforcement until they each completed three 500-trial blocks (the last two consecutively) in which the DR was greater than or equal to 0.80.

#### Probe testing

Following the discrimination phase, all chickadees completed a series of probes and supplemental training described in the following two sections. On probe trials, responses were neither rewarded nor punished: chickadees received a 30-s ITI with the house lights on after responding. For all probe sessions, probe trials comprised ∼20% of all trials. Training sequences during probe sessions were always reinforced as described in the last phase of discrimination training. During each block (∼500 trials, varied by probe session) of probing, all probe sequences and selected *different* sequences from training were presented once (see below for details on the *different* sequences included with each probe). Because there were only 12 trained *same* sequences, these were presented more than once to ensure trained S+ and trained S− sequences occurred equally often. Each trained *same* sequence was presented an equal number of times to the other trained *same* sequences, and the total number of trained *same* sequence trials was equal to the total number of trained *different* sequence trials. In sum, ∼40% of each probe block was made up of trained S+ trials of which 85% were reinforced, ∼40% were trained S− trials punished 100% of the time, and ∼20% were unreinforced probes. After completing a minimum of three blocks (∼1500 trials) on each probe stage, chickadees were returned to the most recently completed discrimination phase (see below for supplemental discrimination phases) for a minimum of one ∼500-trial block with above criteria performance (i.e., a DR ≥0.80). The first three trials with each probe sequence were used for analysis.

#### Novel sequences test 1: Novel orders probe

The purpose of this first probe was to determine whether the chickadees had learned to discriminate *same* from *different* or had instead memorized responses to the 252 fixed *different* sequences used during training. Thus, the order of the *different* sequences was manipulated to present novel sequences of the 12 notes. During this probe, we used all the *same* sequences and a subset of 204 *different* sequences that were presented during training (each of the 12 sounds occurring in each position 17 times). Probe trials consisted of novel sequences of the 12 training stimuli. We tested a total of 96 novel sequences of the original training as probe trials (each of the 12 training stimuli occurred in each position 8 times).

#### Novel sequences test 2: Half and half probe

The purpose of this second probe was to determine whether the chickadees attended to the first or second half of a sequence when responding. To do this, we used probe trials in which either the first half or the second half of a sequence consisted of *same* or *different* stimuli (e.g., 1-1-1-1-1-1-2-3-4-5-6-7 or vice versa). This probe was organized identically to the novel orders probe, except we tested 96 novel sequences where either the first six stimuli within a sequence were the *same* sound (48 sequences, four with each stimulus making up the first half) or the second six stimuli within a sequence were the *same* sound (48 sequences, four with each stimulus making up the second half). All training stimuli were used equally often to make up these probes.

#### Novel stimulus test 1: Pitch constant and timbre constant different probe

The purpose of this probe was to determine: 1) whether the chickadees were memorizing only the *same* sequences and responding by “default” to everything else and 2) whether the chickadees were using either the pitch or timbre dimensions or both dimensions in determining whether a sequence of redundantly-defined sounds was *same* or *different*. Recall the trained stimuli could be distinguished using redundant cues employing both pitch and timbre, so either dimension alone could be sufficient to perform the discrimination. This probe was organized identically to the novel orders probe except that the probes were now composed of novel combinations of pitch and timbre values that were never used in training. We created novel test stimuli from the B3 and F5 pitches at all timbres from training (e.g., only B3 harmonica was included as a training stimulus and so we now included B3 guitar, B3 piano, etc. during testing) and the French horn and harmonica timbres at all pitches from training (e.g., only B3 harmonica was included as a training stimulus and we now included C3 harmonica and D5 harmonica etc.). Using the novel B3, F5, harmonica and French horn stimuli, we created all 44 possible new *same* sequences (e.g., 12 repetitions of B3 guitar) and 48 *different* sequences that differed along the pitch (different pitches/same timbre) or timbre (different timbres/same pitch) dimension (12 of each test type based on B3, F5, harmonica & French horn).

#### Novel stimulus test 2: Single dimension discrimination

At this point, *different* sequences that could only be discriminated on the dimensions of pitch or timbre were introduced into training. Initially, single dimension discrimination was similar to discrimination training except that it used only the novel pitch-only and timbre-only probe sequences from the previous probe sessions. Uni-dimensional *different* sequences were now trained as S+ and novel *same* sequences were trained as S−. For this initial training, all novel S+ sequence trials were reinforced. Chickadees were trained on this uni-dimensional-only stage until six 500-trial blocks (the last two consecutively) in which DR was greater than or equal to 0.80 were completed.

At this point, the original training sequences were re-added and the chickadees trained on this stage until six 500-trial blocks (the last two occurring consecutively) in which DR was greater than or equal to 0.80 were completed. Following this, the percentage of reinforced *different* trials was again reduced to 85% and chickadees were trained until they completed three 500-trial blocks (the last two occurring consecutively) in which the DR was greater than or equal to 0.80.

#### Novel stimulus test 3: Novel timbres probe

The purpose of this probe was to determine whether the chickadees could transfer their S/D discrimination to sequences composed of novel timbres. The baseline discrimination now consisted of using 156 of the original *different* sequences (each note in each serial position 13 times), all the original *same* sequences and all the pitch and timbre uni-dimensional sequences that had previously been trained. The probe stimuli were generated using 12 new timbres (banjo, clavinet, bottle-blow, brass, celesta, sitar, contrabass, dulcimer, atmosphere, fiddle, shakuhachi and voice). Each of these novel timbre stimuli were played at the F5 pitch. We created all 12 possible new *same* sequences (one for each of the new timbres played at F5) and 84 new *different* sequences (each of the 12 new stimuli occurring in each position seven times).

#### Novel stimulus test 4: Novel pitches probe

The purpose of this probe was to determine whether the chickadees could transfer to discriminating *same* and *different* sequences with novel pitches. This probe was identical to novel timbres probe except that the probe sequences were composed of the untrained pitches either within or between (see Table 1 for note names and frequencies) the 3^rd^ and 5^th^/6^th^ octave that were used in training. Twenty-four novel pitch stimuli were created with the familiar alto sax timbre. From these 24 stimuli we created 96 probe sequences: 24 *same* sequences (12 within and 12 between), 36 within *different* sequences (each of the 12 within octave stimuli occurring in each position three times) and 36 between *different* sequences (each of the 12 between octave stimuli occurring in each position three times).

#### Novel stimulus test 5: Complex stimuli probe

The purpose of this probe was to determine whether the chickadees would be able to transfer their established S/D classifications to sounds with more variable and complex harmonic structures. This probe was organized identically to novel pitch and timbre tests except that the sequences were composed from 12 complex stimuli of natural and man-made sound clips (car horn, champagne, drip, electric shaver, lark, machinegun, radio static, spring, tea break, tick tock, typing, water boil; each also standardized to 500 ms with 100 ms between each sound). We used 12 complex *same* sequences and 84 complex *different* sequences (each of the 12 complex stimuli occurring in each position seven times) as probes.

#### Complex discrimination

Initial complex discrimination was similar to discrimination training except that it used only the probe stimuli from the prior complex stimuli probe. Complex *same* sequences were S− and complex *different* sequences were S+. This was done to ensure continued responding by the chickadees to the novel, complex stimuli before exposing them to a second test of complex probes. The percentage of reinforcement for all novel S+ sequences was 100%. Chickadees were trained on this stage until six 500-trial blocks (the last two occurring consecutively) in which the DR was greater than or equal to 0.80 were completed.

At this point, the percentage of reinforcement for S+ sequences was reduced to 85% to prepare the chickadees for the last probe session. Chickadees were trained on this stage until three 500-trial blocks (the last two occurring consecutively) in which the DR was greater than or equal to 0.80 were completed.

#### Novel stimulus test 6: Novel complex stimuli probe

The purpose of this probe was to determine whether the chickadees would be able to transfer the S/D classifications to a novel set of complex stimuli after training with the prior set of complex stimuli. This probe was identical to the previous complex stimuli probe except with a unique set of complex stimuli (each also standardized to 500 ms with 100 ms between each stimulus; bark, bell ting, cappuccino machine, cat, cell phone, church bells, door slam, fireworks, horn, phone, toilet flush, zip). We used 12 complex *same* sequences and 84 complex *different* sequences (each of the 12 complex sounds occurring in each position seven times) as probes.

### Response Measures

To determine whether the chickadees had successfully learned to discriminate *different* S+ sequences from *same* S− sequences we calculated a DR. To calculate the DR, we first calculated the percent responses for S+ and S− sequences, excluding the trials where the chickadee left the perch before the sequence had finished playing. For example, to calculate the percent response for S+ sequences we used the formula R/(T-I) where R was the total number of times the chickadee went to the feeder after hearing an S+ sequence, T was the total number of times an S+ sequence played, and I was the total number of interrupted S+ trials (i.e., chickadee leaving the request perch before the end of the sequence). The identical calculations were made for S− sequences. The DR was then calculated by dividing the percent response for S+ sequences by the sum of the percent response for S+ and S− sequences (note: as a within-chickadee proportional responding measure, the DR eliminates differences in overall frequencies of responding among chickadees). The resulting DR is a value between zero and one where 0 means all visits to the feeder followed S− sequences, 0.5 represents chance with half of the visits to the feeder followed S+ sequences and half followed S− sequences, and 1.0 represents perfect discrimination of S+ from S− trials.

## Results

### Acquisition

To investigate their acquisition of a relational concept, we evaluated first how quickly the chickadees learned the S/D discrimination (i.e., the first time the chickadees were reinforced differentially for *same* and *different* sequences). We examined whether individual chickadees were responding above chance within a trial block by using z-score binomial tests of dichotomous data to determine whether there was significantly more responding following S+ sequences compared to S− sequences. To determine how many blocks were necessary for the chickadees to respond significantly more to *different* than *same* sequences, we performed this calculation across the first 3 blocks of 500 trials of discrimination training. We also analyzed the last block of pretraining as a comparison. Due to performing multiple comparisons, we Bonferroni-corrected our analyses using a p value cutoff of 0.002 (0.05/(6 birds×4 blocks of data).

Overall, the S/D discrimination was acquired rapidly by the chickadees. During their last 500-trial block of pretraining prior to discrimination training, none of the chickadees responded differentially to the future S+ and S− sequences (all *z*s ≤0.36). Within the first 500-trial block of discrimination, once differential reward for *same* and *different* sequence was introduced, three of the six chickadees were responding significantly more to S+ *different* than the S− *same* sequences (all *z*s ≥3.93). By the end of the second block, one additional chickadee was responding significantly more to S+ sequences than S− sequences (*z* = 3.02). The final two chickadees were discriminating significantly above chance by the third block (*z*s ≥9.76). Three chickadees reached the criterion of a DR of 0.80 during the second block of 500 trials, and the remaining three reached this criterion during the fourth block of 500 trials (see Figure 2).

### Novel Sequence Tests

Next we examined how novel sequences created by reordering the 12 training stimuli into novel sequences would be discriminated. Overall, the chickadees continued to accurately perform the S/D discrimination to the trained sequences during the novel sequence tests. Further, as can be seen in Figure 3, novel reordering of the stimuli had little effect on discrimination. We conducted a repeated-measures analysis of variance (RMANOVA) comparing percent response for each sequence type (i.e., trained *same*, trained *different*, novel *different* combinations, 1^st^ half *different*, 2^nd^ half *different*) to determine how responding to order-manipulated probe sequences compared to training sequences. All percentages were logit transformed prior to analysis to control for heterogeneity.

The RMANOVA revealed that there was a significant effect of sequence type on percent response (*F*(4,20)  = 49.91, *p*<0.001). Tukey post-hoc tests showed that all other sequence types had a higher percent response compared to trained *same* sequences (*p*<0.001). There was no difference in percent response to trained *different* sequences and either the novel *different* probes (*p*>0.05), or the 2^nd^ half *different* probes (*p*>0.05). However, the percent response was significantly lower to 1^st^ half *different* probes compared to both trained *different* (*p*<0.01) and novel *different* probes (*p*<0.01), but not 2^nd^ half *different* probes (*p*>0.05). These results indicate that the order of stimuli within the *different* sequences had not been memorized, but instead being processed based on the “different” relations among the notes. Further, responding to these differences was more influenced by the last six stimuli of a sequence than the first six.

### Novel Stimulus Tests

In the novel stimulus tests, we made sequences from stimuli that had not been experienced during discrimination to examine whether the chickadees' S/D discrimination would generalize to novel stimuli. Likely because of their novelty, chickadees' responses to these probes were considerably lower than to the probes containing novel sequences of trained stimuli. The highest percent response for novel stimulus probes for each chickadee ranged from 25–44%, whereas for novel sequence probes it ranged from 66–93%. At the same time, chickadees generally responded more to novel *different* than novel *same* sequences. Considering this, we compared the response to probe “same” stimuli directly to probe “different” stimuli. We did not compare the responses to these probes to the responses to training stimuli. All percentages were logit transformed prior to analysis to control for heterogeneity.

The results of the nine types of novel stimulus tests are presented in Figure 4. Overall, the chickadees showed good S/D discrimination transfer during these tests. In the tests in which only uni-dimensional sequences were tested (i.e., either pitch or timbre was kept constant), chickadees continued to strongly differentiate *different* from *same* sequences for both pitch-only (dependent *t*-tests *t*(5)*s* ≥3.73, all *p*'*s<*0.05) and timbre-only sequences (*t*(5)s ≥3.80, all *p*'*s<*0.05). When tested with novel timbres and novel pitches within the range of their training, the chickadees also exhibited significant S/D discrimination transfer (*t*(5)s ≥3.20, all *p*'*s<*0.05).

In two cases, the chickadees did not show transfer. The first was the novel pitch test, where the pitches were selected from outside the training range (*t*(5)  = 0.80, *p*>0.05). Further, the chickadees also initially failed to show transfer to the first set of complex sounds (*t*(5)  = 2.23, *p*>0.05). Following training experience with complex sounds, however, they did exhibit excellent and significant S/D transfer when subsequently tested with an entirely new set of complex sounds in the second test (*t*(5)  = 2.94, *p*<0.05).

## Discussion

Here we show that black-capped chickadees quickly and accurately learn an auditory S/D discrimination across a wide variety of stimuli. This S/D discrimination transferred without any decrement in performance to novel sequences of trained stimuli. The chickadees also showed robust transfer of this discrimination to all novel stimulus sequences, except for novel pitches that fell into the octave between training stimuli and to their first experience with complex sounds that were completely unlike the single note training sounds. This suggests that black-capped chickadees can readily learn and apply a relational S/D concept to acoustic stimuli.

Taken together with the auditory S/D concept learning demonstrated by pigeons with comparable stimuli [20], [29], and the S/D discriminations demonstrated in budgerigars including variations in pitch and timbre [23], [45]–[48], our results here suggest that the capacity to judge the relations between acoustic stimuli is likely widespread among birds. Because the present experiment involved similar protocols, we can compare the current results with those collected previously in pigeons [29]. For instance, we trained our birds to respond to *different* sequences, rather than *same*, to keep our experiment comparable to the Cook & Brooks [29] study with pigeons. Overall, the pigeons seemed to have more difficulty learning this auditory S/D task than the chickadees. Whereas all chickadees responded differentially within three 500 trial blocks, the pigeons required much more training before they responded differentially to “same” and “different” sequences. In fact, compared to other experiments using synthetic stimuli, all chickadees in the current experiment showed extremely fast acquisition [15]–[18]. Although the differences in performance between the species might be due in part to procedural and response differences between the two studies (e.g., pecking a screen rather than flying to a perch), acoustic stimuli are especially relevant for vocal learning animals like songbirds. Thus, it seems reasonable that the chickadees would have an easier time with this auditory discrimination compared to pigeons (a non-vocal learner). In the end, both species did learn the task and showed very similar patterns of transfers. This suggests a between species difference in attention to the auditory modality rather than conceptual ability. One avenue of future research would be to have the chickadees respond to *same* sequences instead of *different* sequences to see whether this leads to an advantage for this species. Some previous work has found that the chickadees learn a task at similar speeds [49] and responded similarly to probe tests [50] with counterbalanced contingencies, and studies that have not tended to be specifically conspecific versus heterospecific discriminations [39], [51], so we expect that counterbalancing our procedure here would not have a large effect.

As suggested earlier, auditory S/D discrimination could be useful for chickadees, as they need to pay attention to and discriminate among many different vocal signals in the wild. However, it may be that this species' ability to successfully learn S/D discriminations is not specific to the auditory modality but a more general ability. Chickadees not only rely strongly on auditory information because of their complex vocal communication, but visual information as well for seed caching. Black-capped chickadees can cache thousands of seeds daily [52] and remember over long periods where these caches were made [53]. The caches are then re-located by these chickadees using multiple visual cues in addition to spatial cues [54]. In the future it will be interesting to determine whether the capacity exists for S/D discrimination and concept learning in other modalities in chickadees. In pigeons that depend more on the visual domain, compared to the auditory domain, not only have visual S/D relations been shown readily, but the birds learn relational rules to categorize stimuli where either absolute or relational rules could be used [55]–[57]. Thus, the use of relational over absolute strategies may be domain-specific or may be more difficult to achieve outside the primary domain of a given species.

Because of the nature of auditory stimuli, our stimuli had to be presented serially instead of simultaneously as is done in many visual experiments. In previous work looking at serially presented visual displays and S/D learning, Young, Wasserman & Dalrymple [58] showed the later within a sequence that “different” stimuli occurred rather than “same”, the more pigeons would respond to that sequence. The order of presentation also appeared to have a similar impact on the response of the chickadees in the current experiment. The chickadees responded significantly less to sequences in which the last six notes were *same* compared to trained *different* sequences, but this was not true for sequences where the last six notes were *different*. However, in all cases of half *same* and half *different* sequences, chickadees still responded significantly higher than they did to trained *same* sequences. This suggests that chickadees attended to the difference of the first six and the last six notes in each sequence, but weighted the last six notes more heavily than the first six, potentially because they occurred nearer to the one second window the chickadee had to make a decision (go or no/go). By being presented closer to the one-second decision window, it is possible that chickadees' memory for the final six stimuli was more robust. This is not surprising, as recency effects are stronger than primacy effects for serially-presented lists in humans as well [59] (but note that the recency effect may also be modality specific as recency effects are rare in seed caching studies, see [60] for an exception). Further experiments varying the total number of stimuli present within a sequence (e.g., ABCDEFGHIJKL vs. ABCD), the relative number of S/D stimuli within a sequence (e.g. ABCDEFGGGGGG vs. ABCGGGGGGGGG), and the spacing of *same* and *different* stimuli within a sequence (e.g., ABCDEFGGGGGG vs. AGBGCGDGEGFG) could clarify what parameters are necessary for S/D discrimination to occur (see [61] for work with pigeons addressing this issue in the visual domain).

Despite how readily the chickadees transferred their discrimination to the vast majority of novel sounds, it is revealing to examine the novel sounds that were more problematic (i.e., that showed little evidence of transfer of the S/D concept) to understand the limitations. There was very little response to the first probe trials with harmonically-complex sounds, likely because they sounded significantly different from the single notes experienced in training. Chickadees regularly show varying levels of neophobia or a hesitancy to approach the unfamiliar stimuli [62]. Nevertheless, one can see in Figure 4 that, of the responses the chickadees made, the majority were following *different* sequences. Once the chickadees had experience via training with this type of variable and harmonically-complex sound, they showed similar transfer as with single notes when tested with a second set of complex sounds. Interestingly, the pigeons showed a similar pattern of transfer in the experiment we replicated [29]. This suggests that there may be some boundaries or restrictions on relational S/D learning that are established during training based on the breadth of the stimuli experienced. Stimuli within that range can be responded to quite flexibly, but if too far outside that experienced range, animals' ability to use stimulus relations is reduced. Humans show a similar tendency to have more difficulty applying relational rules to novel domains the less alike they are to the original domain in which the rule was learned (e.g., [63]–[64]). In fact, young children first learn to make domain-specific relational judgments to replace characteristic judgments (e.g., understanding that “bigger” refers to the size relationship between two objects rather than a member of the “big” category; [65]).

This “boundary” constraint may explain the lack of transfer to the test involving novel pitches that were produced from the octave between those used in training (i.e., octave four as octave three and five were used in training). That is, stimuli with pitches that were taken from the octave between the training octaves may have been perceived as too far removed from the training stimuli to be categorized with an identical rule. One potential problem with this account is the broad spectra of the notes used in these tests. Because of the use of complex timbres in generating the notes, the spectra of the novel test octave and the training octaves largely overlap. To illustrate this, we have plotted the maximum amplitude across combined stimuli from each octave in Figure 5. The lack of transfer given this overlap suggests instead that the chickadees may have been attending to the fundamental frequency or specific harmonics within the notes rather than the relative frequency structure of their harmonics. Most songbirds studied (see [66]) have extremely accurate absolute pitch compared to mammals, and have difficulty transferring discriminations to novel pitch ranges [12]–[13]. This is generally thought to be because songbirds use absolute pitch rather than relative pitch to classify auditory stimuli [13], although there appear to be exceptions especially when the birds are attending to conspecific vocal cues (e.g., [17], [31], [67]). Because the experiment could not have been solved using absolute pitch alone, but absolute pitch appears to have played a role in their response to the novel pitch probes, the chickadees were likely coding both absolute and relational aspects of the stimuli as pigeons appear to have done in former S/D experiments [20], [55]–[57].

To return to the broader implications of this study, the assumption we made was that if the birds were able to learn a relational task, such as the S/D paradigm we used, that this would support the conceptual-intentional system underlying Hauser, Chomsky & Fitch's [5] model. In turn, this would support the use of songbirds, or at least chickadees, as a model for understanding the difference between human language and other vocal learning systems. Chickadees, as songbirds, are members of one of the most commonly-studied animal groups as a model for language because of the many parallels between their vocal acquisition and production processes and those of human language [6]. By studying additional perceptual, behavioural, and cognitive abilities, besides vocalizations and communication that are relevant for language, we should be able to tease apart what cognitive factors are necessary and sufficient for vocal learning in general, and what commonalities exist among animal groups in cognitive processing with and without vocal learning. In the future, we hope to address both the domain-specificity of our results, and also whether training birds to respond to *same* instead of *different* sequences would generate the same results.

For the comparison between chickadees and humans, the failure of the chickadees to transfer to a novel absolute pitch is especially interesting. Humans tend to have strong relative pitch processing abilities, but strong absolute pitch ability is quite rare in humans [68]. However, there is developmental evidence that suggests that infants have a natural tendency to attend to absolute pitch over relative pitch while adults attend to relative pitch over absolute pitch (e.g., [69]). In addition, absolute pitch abilities are more common in adults that began musical training at a younger age (e.g., [70]–[71]). Moreover, in tonal languages that make use of pitch cues to determine word meaning, absolute pitch is more common [71]. Deutsch, Henthorn, and Dolson [72] also showed that native speakers of tonal languages produce the same word consistently at roughly the same absolute pitch across sessions. On the other hand, native speakers of intonation languages, such as English, do not. Thus it is possible that absolute pitch may simply play a larger role in chickadee vocalizations than in human language, and, through experience, chickadees may learn to rely on absolute pitch features when processing information from vocalizations. In fact, Charrier, Lee, Bloomfield, and Sturdy [33] showed that chickadees do pay most attention to absolute pitch features when identifying notes from their call. That is, they showed that by changing the pitch alone, they could change the note type category that the birds assigned to the note. Given that the chickadees were able to learn a relational S/D discrimination quickly and easily, we believe their heavy reliance on absolute pitch does not take away from their ability to form a relational concept, but further underlines possible structural differences between songbird vocalizations and human language.
